# Diagnosis of gestational diabetes: Are five rounds of blood sampling necessary?

**DOI:** 10.1097/MD.0000000000029025

**Published:** 2022-03-11

**Authors:** Hyun-Hwa Cha, Won Ki Lee, Sujeong Kim, Hyun Mi Kim, Mi Ju Kim, Won Joon Seong

**Affiliations:** a Department of Obstetrics and Gynecology, Kyungpook National University Hospital, Kyungpook National University, School of Medicine, Daegu, Republic of Korea; b Department of Medical Informatics, School of Medicine, Kyungpook National University, Daegu, Republic of Korea.

**Keywords:** 100-g oral glucose tolerance test, 50-g oral glucose tolerance test, gestational diabetes

## Abstract

We aimed to determine the upper and lower cutoff values to simplify the diagnosis of gestational diabetes mellitus (GDM). We investigated the 50-g oral glucose tolerance test (OGTT) results from 1441 pregnancies and identified 423 gravidas who underwent the 100-g OGTT from 2011 to 2019. We collected the results of 50- and 100-g OGTTs. Moreover, we obtained the sum of the 50-g OGTT and 0-hour values, and the sum of those levels and 1-hour values. We determined the upper cutoff at 50-g OGTT, 0-, 1-hour, sum of 50-g OGTT and 0-hour results, and sum of those levels and 1-hour results for the confirmation of GDM. Also, we determined the lower cutoff at these tests for the exclusion of GDM. The upper cutoffs in 50-g OGTT, 0-, 1-hour, the sum of 50-g OGTT and 0-hour were 222, 115, 212, and 315 mg/dL, respectively. The lower cutoffs in 50-g OGTT, 0-, 1-hour, the sum of 50-g OGTT and 0-hour were 131, 65, 151, and 208 mg/dL, respectively. In addition, we discovered that the upper and lower cutoffs in the sum of 50-g OGTT, 0- and 1-hour values were >516 and <373 mg/dL, respectively. We implemented these cutoffs to our study group at 50-g OGTT and 0-, 1-hour of 100-g OGTT. It could omit 2- and 3-hour sampling in 216 gravidas (51.1%). Our approach was able to simplify GDM diagnostic steps in half of our study group.

## 1 Introduction

Gestational diabetes mellitus (GDM) is increasing worldwide with an increase in rate of elderly pregnancy and maternal obesity.^[1]^ The prevalence of GDM in high-risk population increased with pregnancy progression, being 22.4% between 17 and 23 weeks gestation, and reaching up to 60% after 24 weeks in ethnic population at risk for GDM.^[2]^ Prevalence of GDM in general population of pregnant women is reaching almost 10%,^[3]^ while in high-risk patients prevalence is above 25% at 23 weeks of gestations and reaching 30% at 28 weeks of gestation.^[4]^ Despite the diagnosis of GDM is an important component in prenatal care to mitigate adverse perinatal outcomes, the standard method for GDM screening has not yet been established.^[5]^ The two-step approach according to the American College of Obstetricians and Gynecologists is composed of a universal 50-g oral glucose tolerance test (OGTT) followed by diagnostic 100-g 3-hour OGTT, which is performed in South Korea.^[6]^ This method requires five blood samplings that would be very uncomfortable for pregnant women with positive results in the 50-g OGTT. A previous study reported that 12.3% of women at risk of GDM did not complete at least one OGTT, of whom 32.2% never completed testing.^[7]^ Furthermore, it was suggested that a 100-g 2-hour OGTT could serve as an alternative to the 100-g 3-hour OGTT based on the finding that the 2-hour test could detect 93.1% of GDM patients (231 of 248).^[8]^ With this, we tried to simplify these five rounds of blood sampling to three times of sampling in gravidas with positive 50-g OGTT results.

## 2 Patients and methods

We conducted a retrospective diagnostic accuracy study of 1441 pregnant women who had been screened for GDM between 2011 and 2019 at our outpatient clinic. GDM screening was usually performed between 24 and 28 weeks of gestation, and women at high risk for GDM, such as maternal obesity (≥30 kg/m^2^ of body mass index), older age (35 years of age), and history of GDM or macrosomia (≥4.0 kg of birthweight) underwent GDM screening as soon as feasible. GDM screening was repeated between 24 and 28 weeks in high-risk women showing negative results. Gravidas with glycemia after screening 50-g glucose load with values >140 mg/dL were considered screen-positive and underwent a diagnostic 100-g OGTT. Forty-six gravidas with manifesting values of 50-g results between 130 and 139 mg/dL underwent 100-g OGTT based on clinicians’ judgment. We applied the Carpenter and Coustan (C&C) criteria for GDM diagnosis. After distinguishing pregnancies with GDM, we determined the upper cutoffs for the confirmation of GDM (100% of specificity and 100% of positive predictive value, PPV) and the lower cutoffs for its exclusion (100% of sensitivity and 100% of negative predictive value, NPV) in 50-g OGTT, 0-hour value, 1-hour value, sum of 50-g OGTT and 0-hour values, and the sum of those values. Subsequently, we implemented our cutoffs to our study group and observed the number of gravidas that could be omitted beyond 2-hour blood sampling. In addition, we also conducted receiver operating characteristic (ROC) curve analysis to identify the cutoff values for 50-g OGTT, 0-hour value, 1-hour value, sum of 50-g OGTT and 0-hour values, and the sum of those values. We used the IBM SPSS version 21.0 (IBM Corp., Armonk, NY) and MedCalc (MedCalc Software Ltd, Ostend, Belgium) software to develop the cutoffs and ROC curve. Statistical significance was set at *P* < .05. This study was approved by the Institutional Review Board of Kyungpook National University Hospital (IRB file No.: KNUCH-2019-05-003 and date of approval: May 11, 2019). Written informed consent was not given because this study was a retrospective study and used only 50- and 100-g OGTT results.

## 3 Results

A total of 93 out of 423 (22%) of our study group were diagnosed with GDM according to the C&C criteria. The cutoffs for the diagnosis and exclusion of GDM were as follows (Table 1):

1. the upper cutoff for GDM diagnosis in 50-g OGTT was >222 mg/dL,
2. the lower cutoff for GDM exclusion was <131 mg/dL.

**Table 1:** The cutoff values for the diagnosis and exclusion of gestational diabetes mellitus.

Confirmation	50-g (mg/dL)	0-h (mg/dL)	1-h (mg/dL)	50-g + 0-h (mg/dL)	50-g + 0-h + 1-h (mg/dL)
100% of PPV	222	115	212	315	516

Exclusion	50-g (mg/dL)	o-h (mg/dL)	1-h (mg/dL)	50-g + 0-h (mg/dL)	50-g + 0-h + 1-h (mg/dL)
100% of NPV	131	65	151	208	373

NPV = negative predictive value, PPV = positive predictive value.

**Figure 1:**
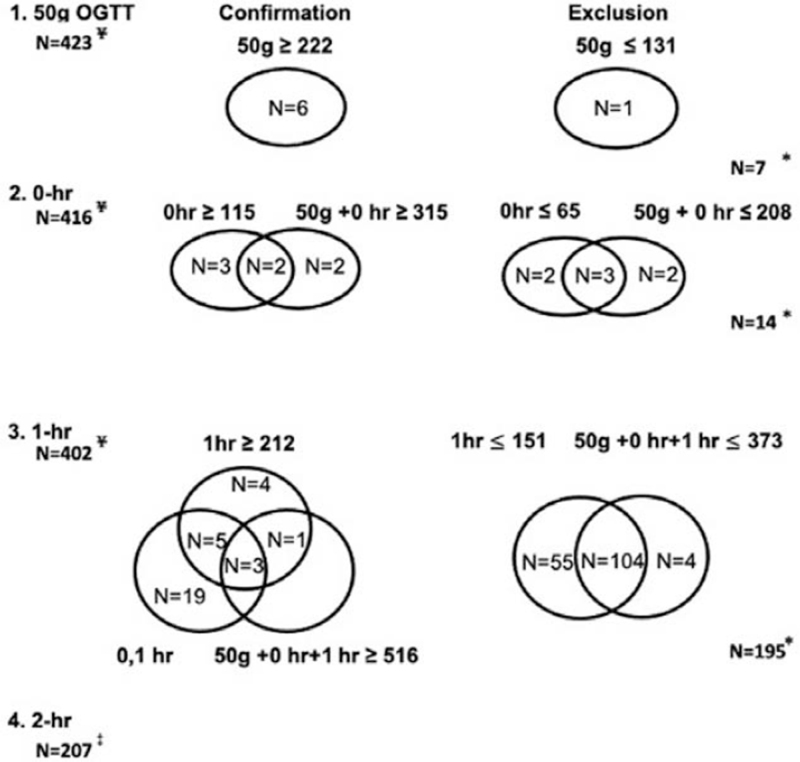
The implementation cutoffs for gestational diabetes mellitus (GDM) confirmation and exclusion at 50-g oral glucose tolerance test (OGTT), 0- and 1-h of 100-g OGTT. ^∗^Number of pregnancies that did not require further evaluation after each step. ^¥^Number of pregnancies which entered this step. ^‡^Number of gravidas who required blood test beyond 2-h.

The upper and lower cutoffs at 0-hour were >115 and <65 mg/dL, respectively. The upper and lower cutoffs at 1-hour were >212 and <151 mg/dL, respectively. Furthermore, the upper and lower cutoffs in the sum of 50-g OGTT and 0-hour value were >315 and < 208 mg/dL, respectively. Moreover, we discovered that the upper and lower cutoffs in the sum of those levels were >516 and <373 mg/dL, respectively. We used a step-by-step approach and phased out pregnant women who did not undergo additional blood sampling. As illustrated in Figure 1, we were able to diagnose or exclude GDM in 216 (51.1%) gravidas without 2- and 3-hour samplings. In conclusion, only 48.9% (207 of 423) of the study group required further testing.

**Figure 2:**
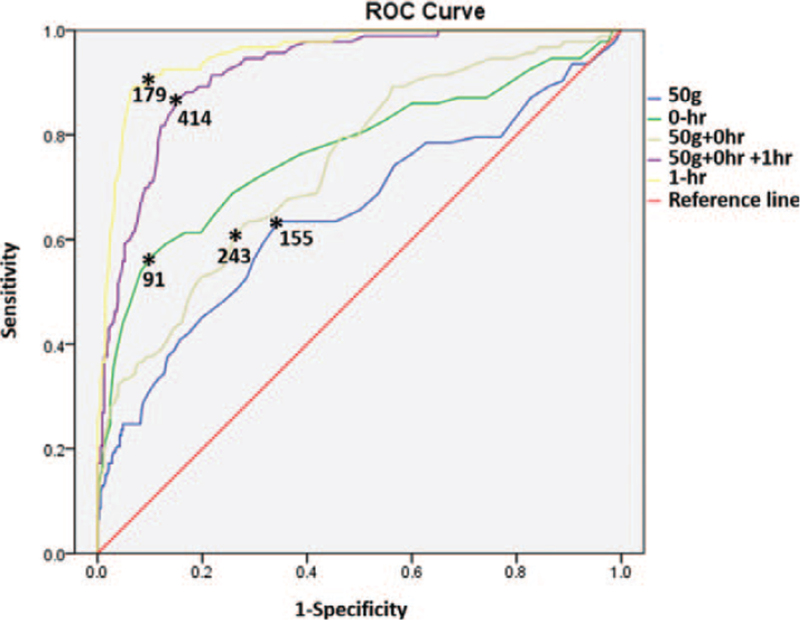
Receiver operator characteristic curve for 50-g glucose tolerance test (OGTT), 0-, 1-h, the sum of 50-g OGTT and 0-h values, and the sum of 50-g OGTT, 0-h and 1-h values.

The ROC curve showed the following optimal cutoffs for GDM diagnosis: 50-g OGTT value of 155 mg/dL, 0-hour value of 91 mg/dL, 1-hour value of 179 mg/dL, sum of 50-g OGTT and 0-hour value of 243 mg/dL, the sum of those values of 414 mg/dL which showed the highest sensitivity and specificity with C&C criteria. Namely, a 50-g OGTT value of 155 mg/dL corresponded to a 63.4% sensitivity and a 65.5% specificity (area under the curve [AUC]: 0.65, 95% confidence interval [CI]: 0.60–0.69, *P* < .001). The 0-hour value of 91 mg/dL corresponded to a 57.0% sensitivity and an 89.4% specificity (AUC: 0.77, 95% CI: 0.73–0.81, *P* < .001). The 1-hour value of 179 mg/dL corresponded to an 89.3% sensitivity and a 93.0% specificity (AUC: 0.96, 95% CI: 0.93–0.97, *P* < .001). The sum of 50-g OGTT and 0-hour value of 243 mg/dL corresponded to a 61.3% sensitivity and a 73.6% specificity (AUC: 0.74, 95% CI: 0.70–0.78, *P* < .001). Lastly, the sum of those values of 414 mg/dL corresponded to an 87.1% sensitivity and an 84.9% specificity (AUC: 0.92, 95% CI: 0.89–0.94, *P* < .001) (Table 2, Fig. 2).

**Table 2:** The optimal values at 50-g OGTT, 0-h, 1-h, sum of 50-g OGTT and 0-h, and sum of 50-g OGTT, 0-h and 1-h by receiver operating characteristic curve.

	Cutoff (mg/dL)	Sensitivity (95% CI)	Specificity (95% CI %)	AUC [95% CI]	*P* value
50-g	155	63.4 (52.8–73.2)	65.5 (60.1–70.6)	0.65 [0.60–0.69]	<.001
0-h	91	57.0 (46.3–67.2)	89.4 (85.6–92.5)	0.77 [0.73–0.81]	<.001
1-h	179	89.3 (81.1–94.7)	93.0 (89.7–95.5)	0.96 [0.93–0.97]	<.001
50-g + 0-h	243	61.3 (50.6–71.2)	73.64 (68.5–78.3)	0.74 [0.70–0.78]	<.001
50-g + 0-h + 1-h	414	87.1 (78.5–93.2)	84.9 (80.5–88.5)	0.92 [0.89–0.94]	<.001

AUC = area under curve, CI = confidence interval.

## 4 Discussion

In this study, our approach was able to confirm or exclude GDM in 216 out of 423 (51.1%) of our study group without 2- and 3-hour blood samplings. We discovered that 45 out of 93 (48.4%) of the GDM cases were determined without 2- and 3-hour blood sampling. Notably, we found that the implementation of our approach could exclude 171 gravidas from GDM without 2- and 3-hour blood samplings. Thus, our results revealed that unnecessary blood sampling to mothers could be minimized.

Several studies have been conducted on GDM screening and diagnosis. These studies usually presented cutoffs of 50-g OGTT that could omit the diagnostic 100-g OGTT. Previous studies on upper cutoffs of 50-g OGTT suggested 185, 220, 228, or 230 mg/dL as the upper cutoff for the omission of 100-g OGTT.^[9–12]^ Consistent with previous results,^[9–12]^ our results also demonstrated that a 222 mg/dL level in 50-g OGTT could be considered the upper cutoff for the omission of 100-g OGTT. Unlike these studies, we tried to simplify the diagnostic step rather than omitting a 100-g diagnostic OGTT. Phaloprakarn and Tangjitgamol also suggested a modified 100-g OGTT consisting of a summation of 1- and 2-hour values, and they reported that the optimal value of this test was 341 mg/dL with 93.5% of sensitivity and 95.2% of specificity.^[13]^ Additionally, previous studies on 100-g OGTT suggested that a 2-hour 100-g OGTT (0-, 1-, and 2-hour) could be a reasonable option based on the result that showed 89.5% to 93.1% of the GDM cases could be detected by 2-hour 100-g OGTT.^[13,14]^ In our study, 39 out of 93 (41.9%) of the GDM cases could be confirmed only by 0- and 1-hour samplings based on C&C criteria. Besides, a total of 146 of 207 (70.5%) of gravidas who were required beyond 2-hour sampling could be diagnosed with or excluded for GDM without 3-hour blood sampling (data not shown). Namely, verifying each result of 0-, 1-, and 2-hour samplings could detect 90.3% (84/93) and exclude 90.7% (107/118) of normal cases from GDM. These results showed that 3-hour blood sampling would be less important in 100-g OGTT; therefore, verifying each result of 100-g OGTT and the omission of later steps of 100-g OGTT could be considered in gravidas showing positive 50-g OGTT results.

Meanwhile, Agarwal et al reported that the 2-hour value was the best predictor for GDM, with an AUC of 0.93 (95% CI: 0.93–0.94), an optimal cutoff of 155 mg/dL, an 83.6% sensitivity, and a 92.8% specificity.^[15]^ They also suggested that using only 0- and 2-hour values may be an alternative diagnostic test in GDM high risk population. However, our data showed that 51.1% (216/423) of our study group could be diagnosed or excluded for GDM before 2-hour sampling. Therefore, we did not present the results of 100-g OGTT beyond 2-hour. Among our approaches, the best AUC showed in 1-hour blood sampling during the 50- and 100-g OGTT. In addition, 1-hour value showed the highest sensitivity and specificity and its addition to the sum of 50-g OGTT and 0-hour values improved both sensitivity and specificity. This result implies the 1-hour value of 100-g OGTT would be the most powerful predictor for GDM diagnosis in our study group. Further studies including a larger study group are warranted to determine which of the 1- or 2-hour value is more useful to confirm GDM.

Recently, there had been an attempt using fasting glucose level to diagnose for GDM. van Gemert et al reported that the using of a fasting glucose of 83 mg/dL or less would miss a third of women diagnosed with GDM.^[16]^ Putoto et al also reported that the omission of 100-g OGTT in women with fasting glucose level <91 could be assessed with three quarters of their subjects.^[17]^ Usually below 90 mg/dL of fasting glucose level is considered as normal value; however, GDM mothers could show postprandial hyperglycemia without fasting hyperglycemia. In our study, the sensitivity of 0-hour was 57.0% and its cutoff for the exclusion was 65 mg/dL, which was low. We thought that the attempt for simplifying of GDM diagnosis should include 1- or 2-hour value.

To the best of our knowledge, this is the first study on lower cutoff values in the 0- and 1-hour 100-g OGTT for GDM exclusion. In terms of the lower cutoff of 50-g OGTT, a value between 130 and 140 mg/dL has been used for the 50-g screening of positive result showing 74% to 83% of sensitivity and 72% to 85% specificity.^[18]^ Furthermore, we tried to present the sum of 50-g results and 0-hour, the sum of these values and 1-hour results of 100-g OGTT for GDM exclusion. As observed, our approach could exclude GDM in 40.4% (171 of 423) of our study group without 2- and 3-hour blood samplings. In addition, the sum of 50-g OGTT results, 0- and 1-hour results could exclude four additional cases from GDM other than normal cases that 151 mg/dL of 1-hour cutoff can exclude.

Our study has several limitations. Compared to previous studies, we included a relatively small number of pregnancies. Not all women with results between 130 and 139 mg/dL in the 50-g OGTT performed a 100-g OGTT. Moreover, we did not consider risk factors for GDM, such as obesity or elderly pregnancy. However, this study aimed to simplify GDM screening in pregnant women rather than stratifying GDM screening according to risk factors. The strength of this study was that it suggested a practical method that can identify gravidas who could omit 2- and 3-hour blood samples.

## 5 Conclusions

GDM was diagnosed or excluded after completion of a 100-g OGTT. However, by applying our method to those who had already started 100-g OGTT, we discovered that 2- and 3-hour blood sampling could be omitted in about half of our study group. Further studies including a larger number of pregnant women could present the optimal method to simplify the GDM diagnosis.

## Author contributions

**Conceptualization:** Won Joon Seong.

**Data curation:** Hyun-Hwa Cha, Sujeong Kim, Hyun Mi Kim, Mi Ju Kim, Won Joon Seong.

**Formal analysis:** Hyun-Hwa Cha, Won Ki Lee, Won Joon Seong.

**Funding acquisition:** Nonremarkable.

**Investigation:** Hyun-Hwa Cha, Sujeong Kim, Hyun Mi Kim, Mi Ju Kim, Won Joon Seong.

**Methodology:** Hyun-Hwa Cha, Won Ki Lee, Won Joon Seong.

**Supervision:** Won Joon Seong.

**Writing – original draft:** Hyun-Hwa Cha, Won Joon Seong.

**Writing – review & editing:** Hyun-Hwa Cha, Sujeong Kim, Hyun Mi Kim, Mi Ju Kim, Won Joon Seong.
